# Comparative plasma proteomics in muscle atrophy during cancer‐cachexia and disuse: The search for atrokines

**DOI:** 10.14814/phy2.14608

**Published:** 2020-10-14

**Authors:** Seongkyun Lim, Kirsten R. Dunlap, Megan E. Rosa‐Caldwell, Wesley S. Haynie, Lisa T. Jansen, Tyrone A. Washington, Nicholas P. Greene

**Affiliations:** ^1^ Cachexia Research Laboratory Exercise Science Research Center Department of Human Health Performance and Recreation University of Arkansas Fayetteville AR USA; ^2^ Exercise Muscle Biology Laboratory Exercise Science Research Center Department of Human Health Performance and Recreation University of Arkansas Fayetteville AR USA

**Keywords:** hindlimb suspension, Lewis lung carcinoma, muscle wasting, PON1, serum amyloid A

## Abstract

Skeletal muscle atrophy is common across a variety of pathologies. Underlying mechanisms of atrophy differ between pathologies, and in many conditions, circulating factors are tied to muscle atrophy. Therefore, we sought to identify alterations to the plasma proteome across divergent forms of muscle atrophy, disuse and cancer cachexia, as potential mediators of atrophy. C57BL6/J mice were assigned to Lewis Lung Carcinoma (LLC)‐induced cachexia, disuse by hindlimb unloading (HU), or control (CON). Plasma samples were submitted for discovery proteomics and targets of interest confirmed by immunoblot. Considerably more peptides were altered in plasma from LLC (91) than HU (9) as compared to CON. Five total proteins were similarly modulated in HU and LLC compared to CON, none reached criteria for differential expression. Serum Amyloid A1 (SAA) was 4 and 6 Log_2_FC greater in LLC than CON or HU, respectively, confirmed by immunoblot. Recent reports suggest SAA is sufficient to induce atrophy via TLR. Therefore, we assessed TLR2,4, and IL‐6 mRNAs in hindlimb muscles. TLR mRNAs were not altered, suggesting SAA effects on atrophy during LLC are independent of TLR signaling. However, we noted > 6‐fold induction of IL‐6 in soleus of HU mice, despite minimal shift in the plasma proteome, indicating potential localized inflammation in atrophying muscle. Furthermore, paraoxonase 1 (PON1) was highly repressed in LLC mice and largely undetectable by immunoblot in this group. Our data suggest SAA and PON1 as potential novel atrokines for cancer cachexia and indicate localized inflammation in atrophying muscles independent of the plasma proteome.

## Graphical abstract

Skeletal muscle atrophy is a debilitating condition associated with increased morbidity and mortality. In this study, bloodborne factors were assessed via plasma proteomics to identify candidates which may contribute to the development of this condition in disuse and cancer cachexia. We identify Serum Amyloid A as a factor heavily induced in cancer cachexia which may contribute to muscle atrophy in the tumor‐bearing state.

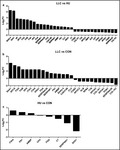

## 1 INTRODUCTION

Muscle atrophy is a pathological dysfunction in protein turnover where an imbalance in protein synthesis and degradation leads to an overall loss in muscle mass and function (Jackman & Kandarian, 2004). Atrophy manifests in response to many diseases and disordered states. These pathologic stimuli trigger divergent responses in skeletal muscle. Chronic inflammation, commonly observed in cancer, sarcopenia, chronic inflammatory disease, and sepsis, preferentially selects fast‐twitch, glycolytic muscle fibers to wasting (Brown et al., 2017; Deschenes et al., 2013; Fearon et al., 2011; Kilsby et al., 2017; Sandri et al., 2004; Singer et al., 2016; Wang & Pessin, 2013). In contrast, conditions of disuse (i.e., denervation, forced bed rest, casting, and space flight), commonly not involving inflammation primarily affect slow‐twitch, oxidative fibers (Bonetto et al., 2012; Brown & Webb, 2018; Brown et al., 2017). Patients afflicted by atrophy experience increased rates of morbidity and mortality while quality of life is significantly reduced (Powers, 2014). Unfortunately, safe and efficacious therapeutic interventions to treat and prevent muscle wasting are lacking.

Two common conditions associated with muscle atrophy are cancer cachexia and disuse. Briefly, cancer cachexia is a multifactorial syndrome associated with an inflammatory microenvironment and characterized by an ongoing loss of skeletal muscle mass, with or without fat loss, that ultimately leads to impaired function (Fearon et al., 2011). Cachexia is currently responsible for approximately 20%–40% of cancer‐related deaths (Fearon et al., 2012; Fox et al., 2009). Because this is a progressive form of wasting that cannot be reversed via nutritional means alone, it is valuable to consider cancer cachexia as a more complex metabolic disorder (Gullett et al., 2011; Porporato, 2016). Similarly, disuse‐induced atrophy, associated with a lack of muscular tension, is a common consequence of forced bed‐rest, limb casting, and space flight (Atherton et al., 2016). Whether temporary or prolonged, disuse can have deleterious effects on force production and can increase morbidity and mortality (Atherton et al., 2016). Both forms of muscle atrophy have been well associated with impairments to rates of muscle protein synthesis and impaired signaling through the canonical insulin‐mTOR cascade mediating protein synthesis (Brown et al., 2018; Shimkus et al., 2018; White et al., 2011). Similarly, in both pathologies multiple markers of elevated protein degradation are elevated through various mechanisms including the ubiquitin proteasome system (UPS), lysosomal proteasome system (LPS) including autophagy, and others (Bodine & Baehr, 2014; Brown et al., 2018; Leermakers et al., 2019).

However, while most forms of muscle atrophy display this shift toward reduced protein anabolism and elevated protein catabolism various other factors differentiate the mechanisms that may induce muscle atrophy. Specifically, cancer cachexia, along with other pro‐inflammatory conditions, commonly exhibits an elevation in chronic systemic inflammation often most notable through the presence of elevated cytokines. Specifically, the Apc^Min/+^ mouse, a commonly used model of spontaneous colorectal cancer and associated cachexia, displays a significant reliance on the cytokine Interleukin‐6 (IL‐6) for the development of cachexia (Baltgalvis et al., 2008, 2009). Interestingly, this reliance upon IL‐6 for development of cachexia in the Apc^Min/+^ model appears to be sex specific, where more recent works demonstrate a lack of IL‐6 dependence in the female mouse (Hetzler et al., 2015). Furthermore, in contrast to the IL‐6 dependence in male Apc^Min/+^ mice, other models of cancer‐cachexia are proposed to be more reliant upon tumor necrosis factor α (TNFα; Chiappalupi et al., 2020; Llovera, et al., 1998; Llovera, et al., 1998). This contrast demonstrates a reliance upon bloodborne factors in at least some forms of muscle atrophy; however, the specific reliance on these factors commonly differs between precise models and biological sexes. These works in cancer‐cachexia suggest the likely and important role of the plasma proteome for development of at least some muscle atrophies.

To the best of our knowledge, plasma proteomic analysis comparing these disparate forms of muscle atrophy (cancer‐cachexia and disuse‐induced atrophy) has not been previously performed. Performing such global plasma proteomic analysis could lead to the identification of novel secretory pro‐atrophic factors (atrokines) and potentially reveal overarching targets linking the two forms of atrophy. Therefore, the purpose of this study was to compare and contrast the plasma proteomes between pre‐clinical models of cancer‐cachexia and disuse‐induced atrophy to identify novel secretory atrokines as potential markers and mechanisms of muscle atrophy.

## 2 METHODS

### 2.1 Animals and interventions

All animal experiments were approved by the Institutional Animal Care and Use Committee of the University of Arkansas, Fayetteville. In total, 34 male and 42 female C57BL/6J mice were purchased from Jackson Laboratories (Bar Harbor, ME). The animals were housed in a temperature‐controlled room and maintained on a 12:12 hr light‐dark cycle. The mice were given ad libitum access to normal rodent chow and water.

In this study, we utilized two different pre‐clinical models of muscle atrophy—hindlimb unloading (HU) to recapitulate disuse‐induced atrophy and Lewis Lung Carcinoma (LLC) implantation to induce cancer cachexia. A cohort of control (CON) animals was age matched to both experimental groups. All animals were singly housed to match housing requirements of HU. This design created three experimental groups: CON, LLC, and HU which were then duplicated to represent both male and female mice. Experiments were performed such that animals in all experimental groups were euthanized and tissue collected at 11–12 weeks of age in order to age‐match groups at time of tissue collection. At the end of designated interventions, animals were anesthetized under isoflurane, body weight recorded and animals humanely euthanized. Plantaris, gastrocnemius, soleus, extensor digitorum longus (EDL), tibialis anterior, quadriceps, spleen, epididymal fat, testes, and plasma were quickly collected post‐euthanasia. Tissue samples were weighed and snap‐frozen in liquid nitrogen for further processing and stored at −80ºC. Plasma samples were centrifuged at 3,500 g for 15 min. Plasma was pipetted into separate sample tubes and stored at −80ºC. One hundred microliterof plasma was aliquoted and sent to the University of Arkansas Medical Sciences TRI Proteomics Core for discovery proteomics (detailed below).

### 2.2 Lewis lung carcinoma culture and implantation

LLC cells were prepared as described previously (Brown et al., 2017). LLC cells (ATCC CRL‐1642) were plated at passage 2. Cells were cultured in 250 ml culture flasks in DMEM supplemented with 10% fetal bovine serum supplemented with 1% penicillin and streptomycin. Once cells reached confluence, they were trypsinized, counted, and diluted in PBS for implantation. Mice were anesthetized with isoflurane and hair was removed from the right hind flank. LLC cells (1x10^6^) suspended in 100 µl sterile PBS and injected subcutaneously into the hind flank of mice at 8 weeks of age as previously described (Brown et al., 2017). Tumors developed for approximately 3–4 weeks. Experimental endpoints were adjusted for signs of distress, veterinary recommendation for humane care, and tumor development in all LLC animals was for at least 21 days.

### 2.3 Hindlimb unloading

Skeletal muscle disuse atrophy was induced by hindlimb suspension as previously described (Washington et al., 2011) with minor modifications. Beginning at 11 weeks of age, mice were subjected to 7 days of hindlimb suspension. Age was selected to approximately age match to the LLC group at time of tissue collection and euthanasia. Mice were acclimated to handling by researchers for one week prior to unloading. Unanesthetized animals’ tails were cleansed with alcohol and betadine, covered with a small amount of benzoin tincture, and dried until the benzoin became tacky. A strip of Tensoplast Tape was wrapped around the base of the tail, just above the hairline. Tape was adjusted so that it did not impede blood circulation and provided an attachment site for the fish‐line swivel device connected to the top of the cage. Mice were attached to a hindlimb suspension apparatus designed to allow access to all areas of the cage with only their forelimbs able to contact the cage floor. The unloading device used in our laboratory consists of a single rod that reaches across the cage. The rod has two rubber stoppers to restrain the mouse from moving too close to the cage walls and loading its hindlimbs. The fish‐line swivel connects to a pulley system on the rod, allowing the mouse to move with minimal resistance along the rod between the rubber stoppers. The mouse was suspended so that the front legs are in contact with the cage floor and the hindlimbs are completely unloaded and unable to reach the floor. The suspension angle was shallow to reduce fluid shifts. The floor was fitted with gridwire to prevent the animal from reloading its hindlimbs with piled up bedding. Animals were suspended for seven days. Animals were anesthetized under isoflurane and body mass was recorded.

### 2.4 Estrus cycle monitoring in female mice

Monitoring of estrus cycle was completed as previously described with minor modifications (Ajayi & Akhigbe, 2020; Caligioni, 2009; Hetzler et al., 2017; McLean et al., 2012). Mice were grasped at the bottom of the neck by skin and restrained holding the tail away from the vaginal canal with the researcher's fifth digit (pinky finger). Once the animal was safely restrained, the vaginal opening was cleaned using a sterile ethanol pad. Then, a specialized micro‐transfer pipet was filled with ~ 50 µl of sterile H_2_O and injected into the vaginal canal. Using the pipet, the sterile H_2_O was flushed into the vagina for collection of vaginal wall cells. The H_2_O/cell mixture was then removed from the vagina on the final flush and the mixture (~50 µl) was dispensed on a microscope slide. Slides were air‐dried overnight to allow for cell adhesion to the microscope slide. Slides were stained with 0.1% crystal violet stain (Sigma Aldrich, St. Louis, MO Cat# C0775; McLean et al., 2012) and visualized using a white light microscope. Stages of the estrous cycle were defined as follows, Proestrus—the presence of predominantly nucleated epithelial cells, Estrus—the presence of predominantly cornified epithelial cell (small or absent nuclei), Metestrus—the presence of leucocyte cells and a mixture of nucleated epithelial and cornified squamous epithelial cells, Diestrus—the presence of predominantly leukocyte cells (Caligioni, 2009; McLean et al., 2012). Vaginal lavages and monitoring of estrous cycle began 14 days prior to any intervention to allow for measurement of baseline estrous cycling as per previous recommendations (Caligioni, 2009; McLean et al., 2012). For CON mice that did not undergo any interventions, estrous cycle monitoring began 14 days prior to the HU intervention to ensure a 14 days baseline to compare against LLC and HU interventions. All samples were collected between 3 and 5 p.m. daily.

### 2.5 Proteomics analysis

Proteomics analysis was completed by the University of Arkansas for Medical Sciences TRI Proteomics Core. Prior to proteomics albumin and other highly abundant proteins were depleted using Pierce Top 12 Abundant Protein Depletion Spin Columns (ThermoScientific, Waltham, MA, USA) as per the manufacturer's instructions. Tandem mass tag (TMT) proteomics was performed off‐site (Porporato, 2016). Once purified from plasma samples, proteins were reduced, alkylated, and digested using filter‐aided sample preparation (Wisniewski et al., 2009). Tryptic peptides were labeled using tandem mass tag isobaric labeling reagents (Thermo) according to the manufacturer's instructions and combined into multiplex same groups. Normalization was achieved by including a pooled reference sample for each group. Labeled peptide multiplexes were divided into 36 fractions on a 100 × 1.0 mm Acquity BEH C18 column (Waters) using an UltiMate 3,000 UHPLC system (Thermo) with a 40 min gradient from 99:1 to 60:40 buffer A:B ratio. This was done under basic pH conditions. Samples were then consolidated into 12 super‐fractions. The super‐fractions were further separated by reverse phase XSelect CSH C18 2.5 µm resin (Waters) on an in‐line 150 × 0.075 mm column using an UltiMate 3,000 RSLCnano system (Thermo). Elution of the peptides was accomplished using a 60 min gradient from 97:3 to 60:40 buffer A:B ratio. Eluted peptides were ionized by electrospray (2.15 kV) followed by mass spectrometric analysis on an Orbitrap Fusion Lumos mass spectrometer (Thermo). MS3 parameters were used for this analysis. MS data were acquired using the FTMS analyzer in top‐speed profile mode at a resolution of 120,000 over a range of 375 to 1500m/z. Upon CID activation with normalized collision energy of 35.0, MS/MS data were acquired using the ion trap analyzer in centroid mode and normal mass range. Up to 10 MS/MS precursors were selected for HCD activation with normalized collision energy of 65.0 using synchronous precursor selection. This was followed by the acquisition of MS3 reporter ion data using the FTMS analyzer in profile mode at a resolution of 50,000 over a range of 100–500 m/z. Protein identification and reporter ion quantification was accomplished using MaxQuant (Max Planck Institute) with a parent ion tolerance of 3 ppm, a fragment ion tolerance of 0.5 Da, and a reporter ion tolerance of 0.01 Da. Scaffold Q + S (Proteome Software) was used to verify MS/MS‐based peptide and protein identifications and to perform reporter ion‐based statistical analysis. Protein identifications were accepted if they could be established with less than 1.0% False Discovery Rate (FDR) and contain at least 2 identified peptides. Protein probabilities were assigned by the Protein Prophet algorithm (Nesvizhskii et al., 2003).

Buffer A=0.1%formic acid,0.5%acetonitrile

Buffer B=0.1%formic acid,99.9%acetonitrile

Both buffers were adjusted to pH 10 with ammonium hydroxide for offline separation.

### 2.6 Pathway analysis

Ingenuity Pathway Analysis (IPA; Qiagen, Valencia, CA; http://www.ingenuity.com) software was used for canonical pathway analysis, upstream analysis, and network discovery.

### 2.7 Immunoblotting

Immunoblotting of plasma was performed as described previously (Brown et al., 2017; Lee et al., 2016; Rosa‐Caldwell et al., 2019). Membranes were probed overnight with primary antibodies. Equal volumes of plasma were loaded to each lane of SDS‐PAGE gels. Protein targets were selected based on the proteomic data provided by the UAMS Proteomics Core Facilities: SOD3 (R&D Systems AF4817), SAA1/2 (R&D Systems AF2948) and PON1 (Novus Biologicals NBP2‐19893). Primary antibodies were isolated from goat. Antibodies were diluted in Tris‐buffered saline, 0.1% Tween 20 with 5% milk. Membranes were imaged on LiCor Odyssey FC using IR detection. Images were analyzed via Alpha View software. All bands were normalized to the 25 kDa band of Ponceau S stain used as a loading control.

### 2.8 RNA isolation, cDNA Synthesis, and quantitative real‐time PCR

RNA isolation, cDNA synthesis, and quantitative real‐time PCR were performed as we have previously described (Brown et al., 2017; Lee et al., 2016; Rosa‐Caldwell et al., 2019). All targets were assayed using Taqman probes including: SAA1 (Mm00656927_g1), TLR2 (Mm00442346_m1), TLR4 (Mm00445273_m1), Trim63/MuRF‐1 (Mm01185221_m1), Fbxo32/Atrogin‐1 (Mm00499523_m1), IL‐6 (Mm0446190_m1), TNF‐α (Mm00443258_m1), and 18S (Mm03928990_g1). All Taqman probes were purchased from Applied Biosystems. 18S Ct values were confirmed to not differ between experimental conditions for each comparison.

### 2.9 Statistical analysis

Phenotypic, immunoblot, and RT‐PCR data were analyzed within each sex with a one‐way ANOVA and Tukey post‐hoc test, α set at 0.05, analyses were performed with SAS software. Proteomic data were analyzed with a one‐way ANOVA, α set at 0.05 using the Q + Quantitation module within Scaffold. Within Scaffold, Benjamini‐Hochberg correction adjusted α to 0.026. For proteomic analysis, differentially expressed proteins (DEP) were thus identified as surpassing threshold levels of *p* < .026 and Log_2_FC = 0.6.

## 3 RESULTS

### 3.1 Muscle atrophy induced by cancer cachexia and hindlimb unloading

**FIGURE 1:**
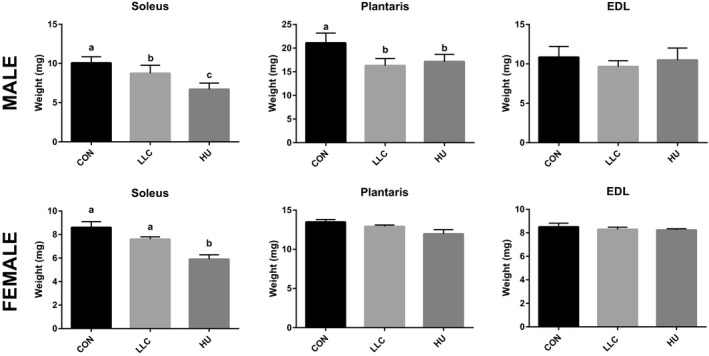
Muscle weights of soleus, plantaris, and EDL in CON, LLC, and HU mice within each sex. All mice are included in the dataset. Lettering denotes statistical significant with alpha set at 0.05

Phenotypic descriptors of applicable body and tissue weights are presented in Table 1. In male mice, when we accounted for tumor mass, total LLC body weight was not significantly different from CON or HU (*p* > .05). Total HU body weight was significantly lower (8%) than CON (*p* < .05). However, tibia length, an indicator of total body size independent of weight, was not different between experimental groups. Therefore, as total body size was not different tissue weights are presented as raw, non‐normalized averages. In both LLC and HU groups, plantaris and gastrocnemius, weights were significantly lower compared to CON (9%–23% *p* < .05). However, these weights were not significantly different between atrophic conditions (*p* > .05). Soleus weight was lower in HU compared to CON and LLC (33.4% and 23.2%, respectively), while LLC soleus weight was only significantly lower compared to CON (13.2%, *p* < .05). Spleen wet weight, a surrogate marker of inflammation, was ~ 200% greater in the LLC animals compared to CON and HU (*p* < .05) (Table 1, Figure 1).

**TABLE 1:** Body and wet tissue weights at time of harvest in control (CON), hindlimb‐unloaded (HU), and Lewis lung carcinoma tumor‐bearing mice (LLC). Lettering denotes statistical significance between groups, alpha set at *p < .05*

	MALE			FEMALE				
Group	CON (*N* = 10)	LLC (*N* = 14)	HU (*N* = 10)	CON (*N* = 10)	LLC (*N* = 23)			HU (*N* = 9)
Body Weight (g)	26.06 ± 0.47a	26.96 ± 0.4 a	23.99 ± 0.46 b	19.56 ± 0.28a		22.09 ± 0.30 b	18.87 ± 0.69a	
Tumor (g)	NA	1.84 + 0.18	NA	NA		2.93 ± 0.23	NA	
Body Weight ‐ Tumor (g)	26.06 ± 0.47a	25.12 ± 0.43a	23.99 ± 0.46b	19.56 ± 0.28		19.16 ± 0.15	18.87 ± 0.69	
Soleus (mg)	10.07 ± 0.25a	8.74 ± 0.27 b	6.71 ± 0.25 c	8.35 ± 0.44a		7.60 ± 0.20a	5.90 ± 0.38b	
Plantaris (mg)	21.09 ± 0.66a	16.31 ± 0.4 b	17.17 ± 0.48b	13.48 ± 0.31		12.90 ± 0.20	12.17 ± 0.63	
Gastrocnemius (mg)	131.51 ± 1.76a	119.9 ± 2.36 b	114.03 ± 2.23b	95.39 ± 2.16a		93.19 ± 1.26a	76.51 ± 2.36b	
EDL (mg)	10.84 ± 0.43	9.66 ± 0.2	10.48 ± 0.48	8.51 ± 0.31		8.30 ± 0.19	8.23 ± 0.11	
TA (mg)	49.41 ± 1.0a	44.15 ± 0.89 b	46.47 ± 0.99a	37.35 ± 0.32a		36.05 ± 0.50a	31.46 ± 1.06b	
Spleen (mg)	69.05 ± 1.89a	164.52 ± 12.07b	63.44 ± 9.97a	75.93 ± 2.22a		293.22 ± 13.97b	54.00 ± 5.12a	
Gonadal Fat (mg)	532.16 ± 39.94a	351.03 ± 16.27b	289.17 ± 20.83b	271.36 ± 27.71a		127.24 ± 16.26b	243.30 ± 25.30a	
Testes (mg)	114.91 ± 2.98	109.82 ± 2.64	113.09 ± 3.46	NA		NA	NA	
Tibia (mm)	17.46 ± 0.04	17.28 ± 0.1	17.49 ± 0.04	16.47 ± 0.06		16.53 ± 0.04	16.39 ± 0.11	
% Acyclic	NA	NA	NA	0		0	0	

In female mice, we observed significantly lower soleus, gastrocnemius, and tibialis anterior masses in HU compared to CON mice (16%–29%, *p* < .05). However, female LLC mice did not display significant differences in muscle masses compared to CON despite significant tumor mass, and compared to CON lower gonadal fat mass (53%, *p* < .05) and elevated spleen mass (285%, *p* < .05, surrogate marker of inflammation). Prior data have demonstrated that female mice typically become cachectic only after becoming acyclic (Counts et al., 2019; Hetzler et al., 2017). Therefore, to account for this we allotted a larger sample size to the female LLC group (*n* = 30) and tested estrous cycle throughout, however, none of the mice in the current study became acyclic in agreement with the lack in measurable muscle wasting in these mice (Table 1). These observations suggest a significant protection against cancer cachexia induced muscle atrophy in female mice. Considering the lack of developed cancer cachexia associated muscle wasting in the female cohort, subsequent analyses would be unable to detect changes in the plasma proteome associated with muscle wasting in these mice, therefore despite efforts we were unable to fulfill the primary purpose of the study in this cohort of mice. As such, all subsequent proteomics and downstream analyses all measures were performed in male mice only.

### 3.2 Global proteomics demonstrates largest differences in LLC‐induced cancer cachexia (Table 2)

**FIGURE 2:**
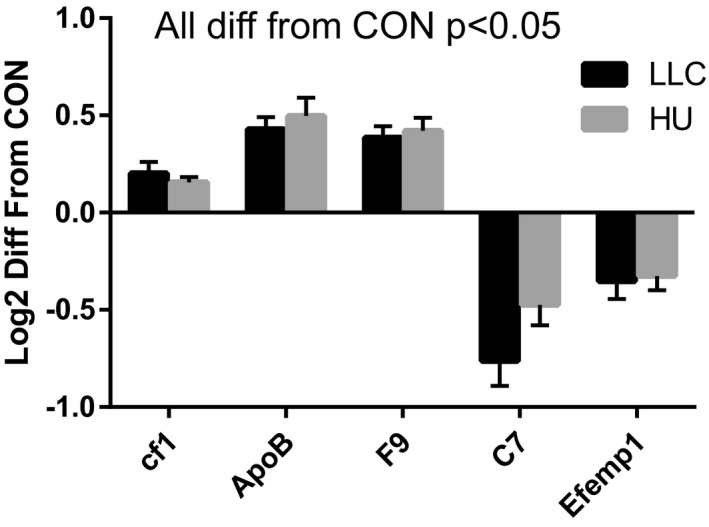
Peptides similarly modulated between LLC and HU compared to CON in male mice. Each of these five peptides reached statistical significance; however, Log_2_FC in HU‐CON did not reach threshold (0.6) to be considered differentially expressed (DE). All peptides significantly different from CON at alpha = 0.05, none different between LLC and HU. All proteomics data were completed with 9–10 samples per group

All proteomics data are deposited in Figshare and can be accessed as Scaffold Viewer file (10.6084/m9.figshare.12994499) or in extracted table (10.6084/m9.figshare.12994511). Using TMT‐based discovery proteomics, a total of 368 Protein IDs were identified in the plasma of the male animals in this experiment. Among these, 5 were similarly modulated between LLC and HU groups and were significantly different compared to CON, though none reached DE thresholds (Figure 2). Of those, three proteins including complement factor 1 (cf1, ~0.2 Log2FC), apolipoprotein B (ApoB, ~0.45 Log2FC), and coagulation factor IX (F9, ~0.4 Log2FC) were higher in plasma from LLC and HU groups compared to CON. Conversely, two proteins were lower in plasma from LLC and HU groups compared to CON including complement 7 (C7, ~−0.76 and −0.47 Log2FC for LLC and HU, respectively) and fibulin‐3 (Efemp1, ~‐0.35 Log2FC). However, while the contents of these peptides were statistically significant, they did not meet the Log_2_FC criterion to be considered DE. No peptides were similar between LLC and HU and met DE criteria (Log_2_FC and statistical significance) from CON. Total number of DE peptides discovered in each comparison of groups are presented in Table 2. The comparison of LLC and HU plasma did yield 104 DE peptides, of which 44 were up‐regulated and 60 were down‐regulated in LLC plasma compared to HU plasma. In comparison of LLC and CON groups 91 DE peptides were observed including: 39 up‐regulated and 52 down‐regulated in LLC compared to CON. In the HU and CON comparison, five up‐regulated and four down‐regulated DE peptides were observed in HU compared to CON; a total of nine DE peptides.

**TABLE 2:** Differentially expressed (DE, statistical significance *p < .026*, Log_2_FC > 0.6) proteins found in each comparison. Number of up‐regulated and down‐regulated DE proteins shown

Comparison	Up‐regulated DE proteins	Down‐regulated DE proteins	Total DE proteins
LLC‐HU	44	60	104
LLC‐CON	39	52	91
HU‐CON	5	4	9

**FIGURE 3:**
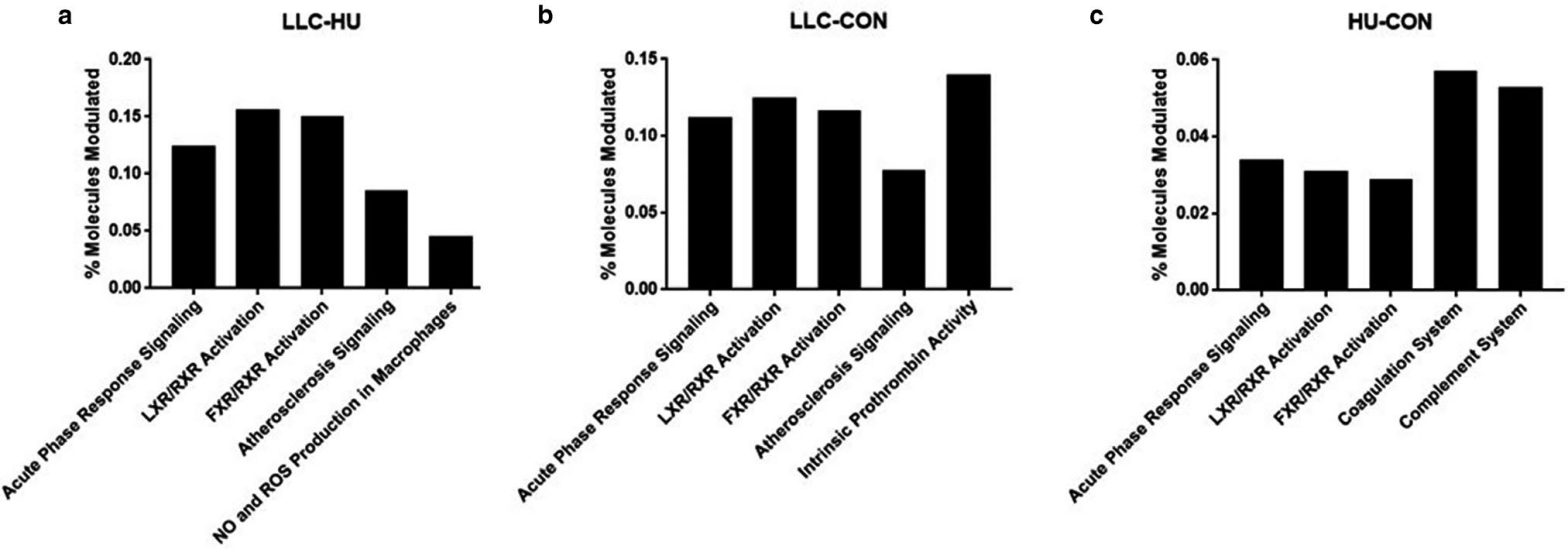
Percent of modulated molecules in the most affected pathways. (a) Modulated molecules in the LLC‐HU comparison. Z‐scores for acute phase response: 3.051, LXR/RXR activation: 0.943, NO and ROS production in macrophages: −1.00. (b) Modulated molecules in the LLC‐CON comparison. Z‐scores for acute phase response: 2.714, LXR/RXR activation: 0.5, intrinsic prothrombin activity: 0.447. C) Modulated molecules in the HU‐CON comparison. Z‐scores for acute phase response: −1.0. All proteomics data were completed with 9–10 samples per group

To determine function and relations between identified peptides we next performed pathway analysis by IPA to determine signaling pathways most affected by shifts in the plasma proteome during muscle atrophy, this analysis was designed to provide insight to the function of identified proteins prior to more detailed examinations. Here we present the top 5 modulated pathways in each comparison (Figure 3), detailed pathways depicting the role of specific peptides identified in this study are shown in Figures S1‐S3 (10.6084/m9.figshare.12994496).

### 3.3 Identification of differentially expressed plasma peptides

**FIGURE 4:**
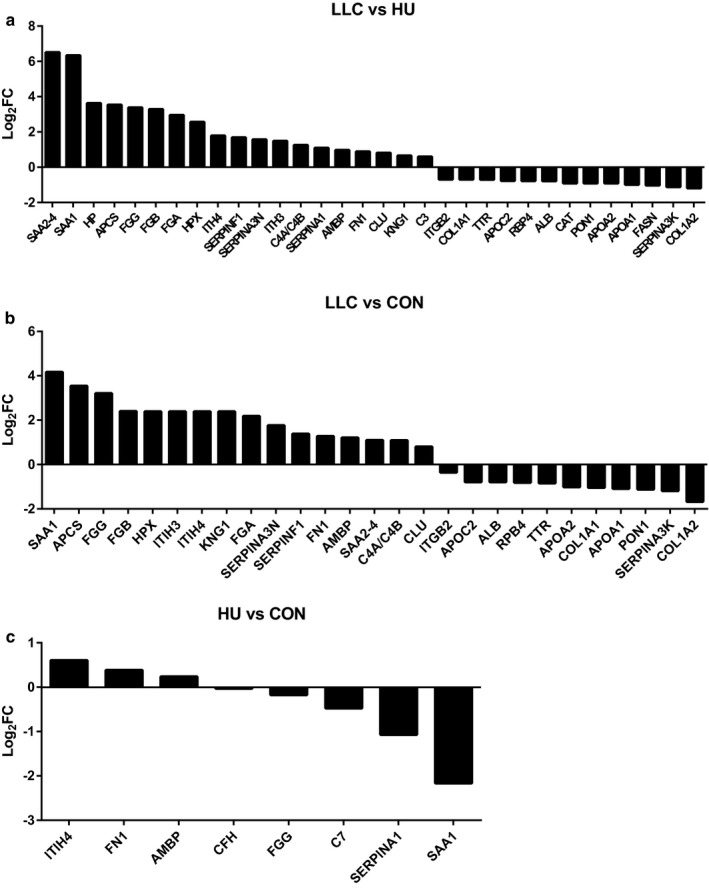
Most up and down‐regulated peptides in each comparison between experimental groups. (a) Most modulated peptides between LLC and HU groups. (b) Most modulated peptides between LLC and CON groups. (c) Most modulated peptides between HU and CON groups. All proteomics data were completed with 9–10 samples per group

In Figure 4, we have depicted DE peptides for each comparison among the most modulated DE peptides observed among modulated pathways shown in Figure 3. Many DE peptides implicated in these modulated pathways exhibit predicted activity across multiple pathways and comparisons. Among this selection 32 DE proteins, 19 up‐regulated and 13 down‐regulated, were involved in the LLC‐HU comparison. Twenty‐seven DE proteins, 16 up‐regulated and 11 down‐regulated, were implicated in the LLC‐CON comparison. Seven DE proteins, three up‐regulated and four down‐regulated, were found in the HU‐CON analysis. The protein Serum Amyloid A (SAA, both SAA1 and SAA2‐4 isoforms), implicated in acute phase response signaling, LXR/RXR and FXR/RXR activation, were involved in every comparison and were the most modulated. Interestingly, SAA responded to atrophy in a divergent manner, being up‐regulated in LLC plasma and down‐regulated in HU plasma (Log_2_FC: 4.17 LLC, −2.16 HU compared to CON). Apolipoproteins, Fibrinogens, SerpinA3, SerpinF1, PON1, antioxidant proteins, and others were found to be predominantly modulated in LLC plasma. Similarly, SAA, SerpinA1, and Fibrinogens were predominantly modulated in HU plasma.

### 3.4 Immunoblot confirmation of plasma proteomics

**FIGURE 5:**
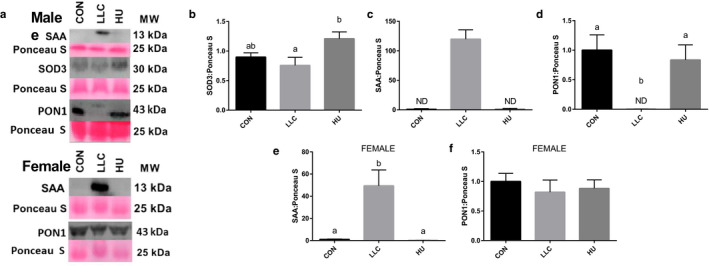
Immunoblot confirmation of SAA1 and SOD3 in plasma of LLC and HU mice. (a) Sample immunoblots for SAA, SOD3, and PON1. (b) Quantitation of SAA content in plasma of male LLC and HU mice. (c) Quantitation of SOD3 content in plasma of male LLC and HU mice. SOD3 was used as a surrogate marker for anti‐oxidants as other anti‐oxidants observed in proteomics exhibit molecular weights which overlay albumin. (d) Quantitation of PON1 content in plasma of male LLC and HU mice. (e) Quantitation of SAA content in plasma of female LLC and HU mice. (f) Quantitation of PON1 content in plasma of female LLC and HU mice. Lettering denotes statistical significance with alpha set at 0.05. For all immunoblot analysis an n of 8–9 samples per group was used

Immunoblot analysis was used to confirm select plasma proteomics findings. Most specifically we performed immunoblot analysis of SAA, PON1, and of Superoxide Dismutase 3 (SOD3). SOD3 was chosen as a surrogate of antioxidant proteins that has previously been observed to be readily induced by physical exercise and to protect against some forms of muscle atrophy ( Okutsu et al., 2014). According to our proteomics data, SAA was highly expressed in LLC plasma and repressed in HU plasma. Strikingly, SAA protein was greater than 100‐fold higher in the plasma from LLC compared to CON or HU mice (Figure 5a,b). Relative to SOD3, proteomics analysis suggested lowered catalase content among other antioxidant proteins in LLC samples compared to HU plasma. SOD3 was probed as an anti‐oxidant surrogate to catalase as the molecular weight of catalase overlays albumin, thereby making the catalase signal indistinguishable from albumin. Immunoblot analysis did not reveal any significant differences in SOD3 content between LLC plasma and other conditions (*p* > .05). However, mean SOD3 content was ~ 60% greater in HU plasma compared to LLC (Figure 5a,c). As a second target altered in LLC plasma by proteomics, we examined Paraoxonase 1 (PON1, >1.1 Log2 FC lower in LLC than CON). Immunoblot confirmation demonstrates PON1 is undetectable in plasma of LLC mice and significantly lower than either CON or HU (Figure 5a,d). Similar to male mice, SAA content was elevated ~ 50‐fold in LLC compared to CON or HU in female mice (Figure 5a,e). Finally, in females we observed no differences in plasma PON1 content between experimental groups (Figure 5a,f).

### 3.5 SAA1/2 induced signaling in skeletal muscles of mice following LLC and disuse‐induced atrophy

First, we assessed SAA1 mRNA in multiple tissues including liver, tumor, spleen, kidney, and lung to attempt to discern where SAA1 may be produced during LLC‐induced cachexia. However, SAA1 mRNA content was very low (Ct values from 38 to not detectable) in spleen, kidney and lung. In liver, despite high expression (Ct ~ 21) no induction was observed in LLC mice. SAA1 expression was observed in LLC tumors with a Ct mean of 35. Due to the lack of significant effects these data are not shown.

**FIGURE 6:**
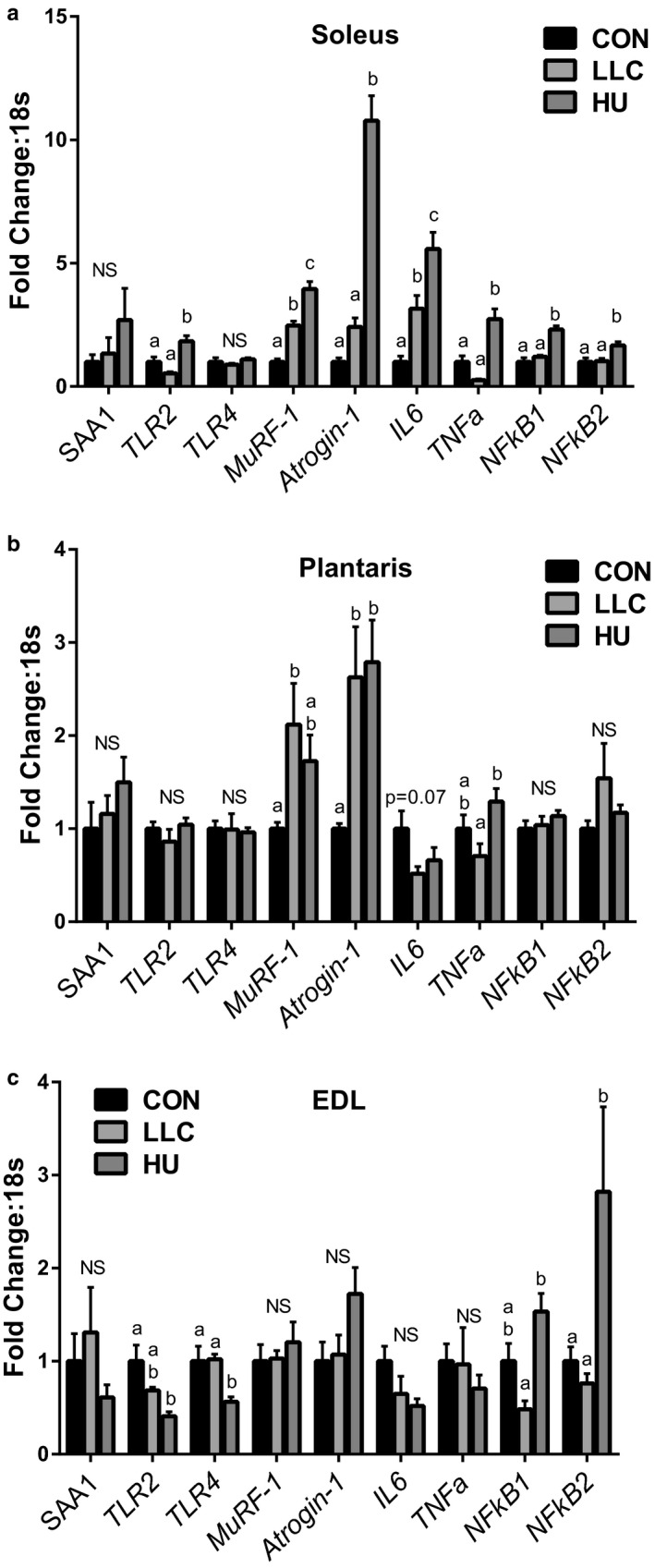
Gene contents of SAA1, TLRs, E3 Ligases, inflammatory cytokines and NFΚB in soleus, plantaris, and EDL muscles. (a) Gene contents in soleus muscle. (b) Gene contents in plantaris muscle. (c) Gene contents in EDL muscle. Lettering denotes statistical significance with alpha set at 0.05. For all RT‐PCR analysis an n of 8 samples per group was used

A recent work by Hahn et al. (Hahn et al., 2020) demonstrated that myotube treatment with recombinant SAA1 can induce atrophy which appears to work through Toll Like Receptor (TLR) signaling, specifically via TLR2 and TLR4. This provides prior evidence of a role of SAA in muscle atrophy. Therefore, in our model we chose to test whether elevated SAA levels in the plasma of LLC mice were associated with similar inductions of gene expression of *SAA1*, *TLR2*, *TLR4*, *MuRF‐1*, *Atrogin‐1*, *IL‐6,* and *TNF‐α* in the muscle as previously observed (Hahn et al., 2020). To do this we tested mRNA contents across soleus, plantaris, and EDL muscles in all three experimental groups, these muscles were selected due to their varying phenotypes (soleus—oxidative, plantaris—mixed fiber, and EDL—glycolytic). We expected that if SAA was impacting muscle atrophy via TLR2/4 signal we would observe inductions in the LLC group only. More so, by testing multiple muscles we were able to test two muscles which exhibited significant atrophy by mass (soleus and plantaris) and one muscle which did not exhibit atrophy (EDL) (Table 1; Figure 1). First, to determine if SAA1 induction was occurring from the muscle itself we tested mRNA of *SAA1* across these muscles finding no significant differences between groups (Figure 6). Interestingly, no significant induction of either *TLR2* or *TLR4* was observed in any of the muscles tested in the LLC group, though *TLR2* content was greater in the soleus of HU mice was seen (~80% greater, Figure 6). As expected with atrophying muscle *MuRF‐1* and *Atrogin‐1* mRNA contents were higher in both LLC and HU mice compared to CON in the soleus and plantaris (1.7 – 10.7 fold) but not in the un‐atrophied EDL (Figure 6). Of note, the largest induction of these targets was observed in the soleus of HU mice (4‐ and 10.7‐fold for *MuRF‐1* and *Atrogin‐1*, respectively) which also displayed the greatest degree of muscle atrophy (Figure 1). As SAA treatment was observed in the work by Hahn et al. (2020) to induce *IL‐6* and LLC has been previously associated with TNF‐α we tested content of these mRNAs. While in plantaris and EDL there was no significant induction of mRNA of either cytokine in LLC or HU compared to CON, induction of *IL‐6* was observed in soleus of both LLC (3.4‐fold) and HU (>6 fold) compared to CON as well as of *TNF‐α* in the soleus only (2.7‐fold). To further assess TLR/NFΚB signaling we assessed mRNA contents of *NFΚB1* and *NFΚB2*. Both isoforms of *NFΚB* were elevated in soleus of HU mice, by 2.3 and 1.7‐fold, respectively, similar to *IL‐6* and *TNF‐α*, with no significant effects observed in LLC mice.

## 4 DISCUSSION

We set out to define the plasma proteome across distinct forms of muscle atrophy, cancer cachexia and disuse, to identify atrophy promoting factors (atrokines) across muscle wasting. We demonstrate large shifts in the plasma proteome in mice undergoing cancer cachexia with few shifts in plasma peptides under conditions of disuse. Specifically, we note the large induction of SAA1/2 in the plasma of tumor bearing mice. Notably, SAAs are sufficient to induce muscle atrophy ( Hahn et al., 2020). Second, we observe the repression of PON1 in plasma of the LLC mice. Our findings suggest SAAs and/or PON1 may provide novel mechanisms of cancer cachexia.

### 4.1 Characterization of cachexia and disuse‐induced atrophy

We set to test the plasma proteome of male and female mice undergoing LLC‐induced cancer cachexia or HU‐induced disuse. While both models induced muscle atrophy in male mice we did not observe significant muscle atrophy in LLC tumor bearing female mice. Prior reports suggest female mice often do not develop cancer cachexia unless they become acyclic (Counts et al., 2019; Hetzler et al., 2017). Therefore, we utilized an increased number in our female LLC cohort seeking develop four groups: CON, HU, Cyclic LLC, and Acyclic LLC. However, no female mice became acyclic or cachectic. This suggests potent protection from cachexia‐induced muscle loss in female mice, should be considered in future studies of cancer cachexia and may provide protective mechanisms for prevention of cachexia. However, due to lack of cachectic female mice we were unable to assess the plasma proteome in female mice following various forms of muscle atrophy.

### 4.2 Identification of alterations in the plasma proteome

Through global proteomics we identified 368 peptides in the plasma. Most strikingly, using established DE criteria, 91 peptides were different in plasma of LLC mice compared to CON (104 compared to HU). In contrast, only nine peptides reached criteria to be considered DE when comparing HU to CON. This demonstrates the differential reliance of the two forms of muscle atrophy on the plasma proteome. Interestingly, though no peptides were similarly altered between LLC and HU conditions compared to CON which reached the DE threshold, five peptides were similarly altered between conditions and met the statistical significance requirement. While purely speculative at this point these five peptides (Figure 2) may provide critical overlap between these forms of muscle atrophy. To provide functional insight, we performed pathway analysis by IPA, which can be reviewed in Figure 3 and supplemental materials.

### 4.3 Serum amyloid A as a potential atrokine in Cancer‐Cachexia

SAA1 was first described as an inducible factor in plasma during cancers in 1993 (Andersson et al., 1993). We observed large shifts in SAA1 and SAA2‐4 in plasma of LLC mice, which was confirmed by immunoblot. While plasma SAAs have been noted in rodent (Noguchi‐Sasaki et al., 2016) and human cancers (Ghweil et al., 2020; Ignacio et al., 2019; Lin et al., 2019; Sun et al., 2019) they have largely not been tied to cachexia. Hahn et al. (2020) demonstrated that recombinant SAA1 induced atrophy in cultured myotubes and muscle atrophy induced by cecal ligation and puncture (CLP) was associated with SAA and TLR/NFΚB induction. In those studies, recombinant SAA1 media concentrations of 10 μg/mL were used to treat C2C12 myotubes, this corresponds to reported SAA1 concentrations in plasma of tumor‐bearing mice (Buczek et al., 2018) and non‐small‐cell lung cancer patients (Kim et al., 2015). Interestingly, despite the lack of cachexia in female mice, SAA was elevated in plasma of female LLC mice. This may suggest SAA primarily as a biomarker of cancer. However, considering the work by Hahn et al. (2020) the induction of SAAs in plasma of LLC mice, SAA cannot be dismissed as a potential mechanism of cancer‐cachexia.

Next, we asked two questions regarding SAAs: (a) where are they produced in LLC tumor‐bearing mice?, and (b) is there evidence SAAs are working through TLR‐induced signaling to promote muscle atrophy in tumor‐bearing mice? To answer the first question we assessed SAA mRNA across tumor, lungs, liver, spleen, kidney, and muscle. Across these tissues we observed no induction of SAA in the LLC mice. However, SAA was expressed by the tumor (Mean Ct ~ 35), a source absent in CON and HU mice. We should do note a prior micro‐array analysis by Bonetto et al. (Bonetto et al., 2011; Hahn et al., 2020) observed induction of SAA in muscles of C26 tumor‐bearing mice, confirmed by immunoblot in quadriceps and gastrocnemius muscle. However, based on our current data we must speculate that SAA is functioning primarily as a tumor‐derived factor.

Next, to assess if SAAs were functioning via TLR‐signaling we examined gene contents of *TLR2*, *TLR4*, *MuRF‐1*, *Atrogin‐1*, *NFΚB1/2, IL‐6,* and *TNF‐α* in each of three muscles. This strategy was in keeping with the observations of Hahn et al. (2020) of induced gene expression following myotube treatment with recombinant SAA. However, we did not observe induction of *TLR2/4,* nor *NFΚB1/2* suggesting if SAA is promoting atrophy in tumor‐bearing mice it is likely not via TLR signaling. In these analyses we did note one final observation of interest. Specifically, induction of IL‐6 and TNF‐α in soleus of HU mice. Disuse is typically not associated with systemic inflammation. However, based on this observation disuse may associate with localized inflammation to affected muscle as observed here by induction of these cytokines. This observation warrants further study as a mechanism of muscle wasting in disuse atrophies.

### 4.4 Paraoxonase 1 as a potential atrokine in Cancer‐Cachexia

Second, we examined PON1, PON1 protects from LDL oxidation and therefore serves an anti‐oxidant function. This becomes very intriguing for a couple of reasons. First, in inflammatory muscle atrophy conditions, that is, cancer cachexia, oxidative stress is a significant known mechanism. In fact, prior work from Okutsu et al. shows extracellular superoxide dismutase (ecSOD) is sufficient to protect from atrophy induced by dexamethasone or calsequestrin expression induced heart failure (Okutsu et al., 2014). While, we previously observed that mitochondrial ROS emission is elevated twofold at the onset of the tumor‐bearing state in LLC mice (Brown et al., 2017). However, effective approaches to ameliorate such oxidative stress in cachexia are not known, thus PON1 may present a novel target of interest. This said, little is known about the role of PON1 and LDL oxidation on muscle, one prior report suggests PON1 deficiency in vivo may exacerbate plasma oxidative stress and high fat‐induced insulin resistance with multiple effects on muscle insulin signaling (Koren‐Gluzer et al., 2013). While LDL oxidation impairs cell growth via induction of mTOR signaling inhibitor REDD2 in macrophages (Cuaz‐Pérolin et al., 2004). Most importantly, we repeated this immunoblot using plasma from our female mice wherein LLC mice did not become cachectic. In that cohort we see no significant difference in PON1 content in the plasma. This contrast in the loss of plasma PON1 in cachectic males, but not in noncachectic females may suggest maintenance of PON1 as a protective mechanism from cachexia in the female mouse. These findings are suggestive of a potential protective effect of PON1 on the muscle phenotype. Therefore, further studies should assess whether PON1 may be a viable target to protect against cancer cachexia.

### 4.5 Summary

In summary, in the current study we sought to identify and compare the plasma proteome across distinct forms of skeletal muscle atrophy in male and female mice. First, in our phenotypic analysis of the female mice in this study we see a potent protection from cachectic muscle loss. This observation bears consideration for further work in the cachexia field and may provide insight to mechanistic targets than can be used to protect from this condition. Second, plasma proteomic responses to cancer cachexia and disuse atrophy vary greatly in degree as only few peptides are altered in disuse while many are impacted during cancer cachexia. Among these we identify induction of plasma SAAs and reduction of plasma PON1 as potential new atrokines in cancer cachexia. Specifically, the maintenance of PON1 in non‐cachectic female LLC mice suggests loss of PON1 as a potential mechanism for the induction of cachectic muscle loss which should be further explored. However, mechanism of action for these targets needs to be further defined. Finally, while not the primary purpose of this study, we not disuse atrophy was associated with localized induction of cytokines IL‐6 and TNF‐α in the most affected muscles (soleus) which may suggest a role for localized inflammation during disuse inflammation.

## DISCLOSURES

The authors declare no conflict of interest.

## AUTHOR CONTRIBUTIONS

NPG and TAW conceived and designed the research. SL, KRD, MERC, WSH, LTJ, TAW, and NPG performed the experiments. SL and KRD analyzed the data. SL, KRD, and NPG interpreted the results of the experiments. SL, KRD, and NPG prepared figures. SL, KRD, and NPG drafted the manuscript. SL, KRD, MERC, WSH, LTJ, TAW, and NPG edited and revised the manuscript. SL, KRD, MERC, WSH, LTJ, TAW, and NPG approved the final version of the manuscript.
